# To: Acute kidney injury and intra-abdominal hypertension in burn patients in intensive care

**DOI:** 10.5935/0103-507X.20190022

**Published:** 2019

**Authors:** Gabriela Cerqueira Caldas Pinto, Michelle Campos Zaupa, Eduardo Juan Troster

**Affiliations:** Intensive Care Unit, Instituto para Tratamento do Câncer Infantil, Hospital das Clínicas, Universidade de São Paulo - São Paulo (SP), Brazil.; Intensive Care Unit, Hospital Municipal Menino Jesus - São Paulo (SP), Brazil.; Intensive Care Unit, Instituto para Tratamento do Câncer Infantil, Hospital das Clínicas, Universidade de São Paulo - São Paulo (SP), Brazil.

**To the Editor**

The difficulty of managing fluid in severely burned patients and the fact that monitoring intra-abdominal pressure (IAP) is not yet a routine intensive care therapy makes the article by Talizin et al.^(1)^ mandatory reading, especially for professionals working with this group of patients.

The systemic inflammatory response observed in severely burned patients is consistent with acute intestinal distress syndrome, a modern theory described by Malbrain that explains the pathophysiology of intra-abdominal hypertension/abdominal compartment syndrome. According to Malbrain, any lesion of ischemia and reperfusion can lead to systemic inflammatory response syndrome (SIRS) and inflammatory intestinal syndrome, which generate increased intestinal vascular permeability. As a result, bacterial translocation, endotoxin absorption, and interstitial edema occur. This edema is responsible for the obstruction of lymphatic drainage from the abdominal cavity, which causes elevation of the IAP and, in turn, more intense SIRS, thus closing the "vicious cycle" of acute intestinal distress.^(2)^

Kidney failure is an important consequence of intra-abdominal hypertension, and its causes are multifactorial, as described by Doty et al.^(3,4)^ in experimental studies with animals. Doty et al. demonstrated that in the group with an isolated increase in renal parenchymal pressure, there were no significant reductions of renal flow and the glomerular filtration rate (GFR), as there was no increase in renin or serum aldosterone levels.^(3)^ However, in the animal model of abdominal compartment syndrome, both GFR reduction and activation of the renin-angiotensin-aldosterone system were observed, which was confirmed by the increased serum angiotensin II and aldosterone. An attempt to correct the cardiac output with volume expansion did not result in reversion of renal dysfunction; however, reversal of the abdominal compartment syndrome quickly reversed the condition.^(4)^

We do not understand why Talizin et al.^(1)^ investigated the use of glycopeptides as a risk factor for intra-abdominal hypertension because we did not find this correlation in the literature. Considering that sepsis and intra-abdominal infection are described by WSACS - The Abdominal Compartment Society as risk factors for intra-abdominal hypertension/abdominal compartment syndrome, we believe that a better characterization of the group exposed to glycopeptide use is required.

The intermittent measurement of intravesical IAP among adults was first described by Kron et al.^(5)^ and was criticized for requiring that the originally sterile indwelling urinary catheter system be opened for instillation of saline for IAP measurement. This technique was then modified by Cheatam and Safcsak,^(6)^ who suggested puncture of one of the indwelling urinary catheters with a Jelco number 18 to decrease the risk of system contamination. This technique, which was used in the study by Talizin et al.,^(1)^ was considered standard by the WSACS in 2013.^(4)^ We disagree, therefore, that the AbViser(tm) anti-reflux valve allows continuous measurement of IAP, as described in the methodology.

The publication of the first consensus with definitions and risk factors for intra-abdominal hypertension and abdominal compartment syndrome by WSACS in 2006^(7)^ and the subsequent data update in 2013^(8)^ are important milestones for the monitoring and management of severely ill patients; however, new studies such as this are needed to prove and highlight the need for monitoring IAP as a vital sign in intensive care.

*Gabriela Cerqueira Caldas Pinto* *Intensive Care
Unit, Instituto para Tratamento do Câncer Infantil, Hospital das
Clínicas, Universidade de São Paulo - São Paulo (SP),
Brazil.* *Michelle Campos
Zaupa* *Intensive Care Unit, Hospital Municipal Menino
Jesus - São Paulo (SP), Brazil.* *Eduardo Eduardo
Troster* *Intensive Care Unit, Instituto para Tratamento
do Câncer Infantil, Hospital das Clínicas, Universidade de
São Paulo - São Paulo (SP), Brazil.*
